# Individual Characteristics in the Comprehension of Pandemic Video Communication: Randomized Controlled Between-Subjects Design

**DOI:** 10.2196/48882

**Published:** 2024-12-04

**Authors:** Daniel Adrian Lungu, Jo Røislien, Ionica Smeets, Siri Wiig, Kolbjørn Kallesten Brønnick

**Affiliations:** 1 SHARE – Centre for Resilience in Healthcare Department of Quality and Health Technology, Faculty of Health Sciences University of Stavanger Stavanger Norway; 2 Science Communication and Society Institute of Biology Leiden University Leiden Netherlands

**Keywords:** video communication, COVID-19 pandemic, comprehension, health literacy, video, videos, health communication, psychology, perception, comprehend, understanding, coronavirus disease 2019

## Abstract

**Background:**

Video played an important role in health communication throughout the COVID-19 pandemic. It was used to communicate pandemic information to the public, with a variety of formats, presenters, and topics. Evidence regarding the effectiveness of video features is available, while how individual characteristics of recipients influence communication comprehension is still limited.

**Objective:**

This study aimed to test 6 individual characteristics and assess their effect on the comprehension of pandemic video communication.

**Methods:**

Short health communication videos were presented to a large sample of subjects, receiving questionnaire responses from 1194 participants. Individual characteristics consisted of age, sex, living area, education level, income level, and belief in science. Communication comprehension consisted of both perceived and objective comprehension. The data were analyzed by multiple linear regression.

**Results:**

Age had a negative effect on both perceived and objective comprehension—age was negatively associated with comprehension. There were sex differences, with higher perceived comprehension and lower objective comprehension among female than male individuals. Living in an urban or a rural area had no significant effect (all *P*>.05). The level of education and income had a positive effect on both subjective and objective comprehension. Finally, the belief in science had a positive effect on perceived comprehension (*P*<.001) but did not have a statistically significant effect on objective comprehension (*P*=.87).

**Conclusions:**

The main differences between those who think they understand pandemic communication and those who comprehend it better are sex (female individuals have a higher perception of having comprehended, while male individuals have higher levels of objective comprehension) and belief in science (higher belief in science leads to higher perceived comprehension, while it does not have any impact on objectively understanding the message conveyed).

## Introduction

### Pandemic Video Communication

A pandemic is a comprehensive public health concern, and effective communication is key to handling the challenges. Unsurprisingly, the COVID-19 pandemic spawned massive efforts from authorities worldwide to communicate a wide range of important information to the public. Effective communication is essential for reducing anxiety and fear in the population [1,2]; building trust in authorities [3,4]; disseminating information and addressing misinformation [5-7]; and encouraging behavioral changes such as social distancing [8,9], hygiene practices, and vaccination [8-10].

Although COVID-19 communication occurred through all media channels, video was among the most used [11-13]. Some videos were developed by governmental agencies, whereas others were developed by public and private media, enterprises, and individual users. While some videos were distributed through traditional media, a massive amount was distributed on social media (eg, TikTok, YouTube, and Facebook) and instant messaging platforms [14-17]. A previous study identified major differences between COVID-19 videos created by public health authorities and professional creators, identifying a creative gap and a limited reach of the videos created by public health authorities [18].

Besides the reach, comprehension is one of the main aims of pandemic video communication, to ensure that the message is conveyed to the population. In a large randomized controlled trial, Vandormael et al [19] reported an increase in knowledge of a COVID-19 animated video distributed through social media. A study from Tan et al [20] found that the quality of COVID-19 vaccine videos distributed on video-sharing platforms was generally low and led to limited comprehension, with slight variations across the platforms.

### Recipients’ Characteristics

On the other hand of the communication process, there is the public, also called recipients of the message. A recent scoping review identified available evidence regarding the effect of recipients’ individual factors on health communication outcomes [21], highlighting the importance of the following 6 factors: age, sex, living area, education, income, and belief in science. Evidence is mixed for the sociodemographic variables, ranging from positive to negative to no effects of age [14,17,22-26], sex [14,19,23,26-29], living area [30-33], education [22,23,26,34-39], and income [34,36,40] on health communication outcomes. Instead, belief in science was found to have a positive effect on health communication outcomes. According to Farias et al [41], belief in science can help individuals to cope with stress and anxiety, similar to religious belief. Research has found that belief in science influences physical distancing behavior: there was less compliance to physical distancing in countries with less belief in science [42] or mask wearing, and higher belief in science predicted higher mask wearing in public spaces [43] during the COVID-19 period.

Studies have identified some features of effective pandemic video communication [7,18,44], but the gap persists regarding the role that individual characteristics of recipients play in the comprehension of pandemic video communication. This study aims to fill this gap by assessing the effect of 6 individual factors (age, sex, living area, education level, income, and belief in science) on the comprehension of pandemic video communication.

The relevance of this study derives from the fact that while video communication factors (eg length, visuals, tone, etc) can be manipulated to increase effectiveness, the individual characteristics of pandemic video communication recipients cannot, and therefore public health authorities should be aware of potential differences when developing pandemic communication videos.

## Methods

### Design and Video Creation

This study analyzed individual characteristics from a trial using a full-factorial, between-subjects randomized controlled experiment [45]. The design of the videos was done by manipulating 3 variables: the source, the topic, and the call to action.

The source had two levels: (1) in one case, the presenter was introduced as an infectious diseases specialist, and (2) in the other, he was presented as a salesman. We chose 3 topics to communicate: exponential growth, handwashing, and the burden of pandemics on health care systems. The call to action had two levels: one with a motivational call to action at the end of the video, and one without. The design led to 12 video versions to be created. A professional scriptwriter was hired to create 6 scripts (3 topics with 2 tones), while the third variable was manipulated through the on-screen text introducing the speaker. After 2 rounds of revision, the scripts were approved by the researchers and the writer.

**Figure 1:**
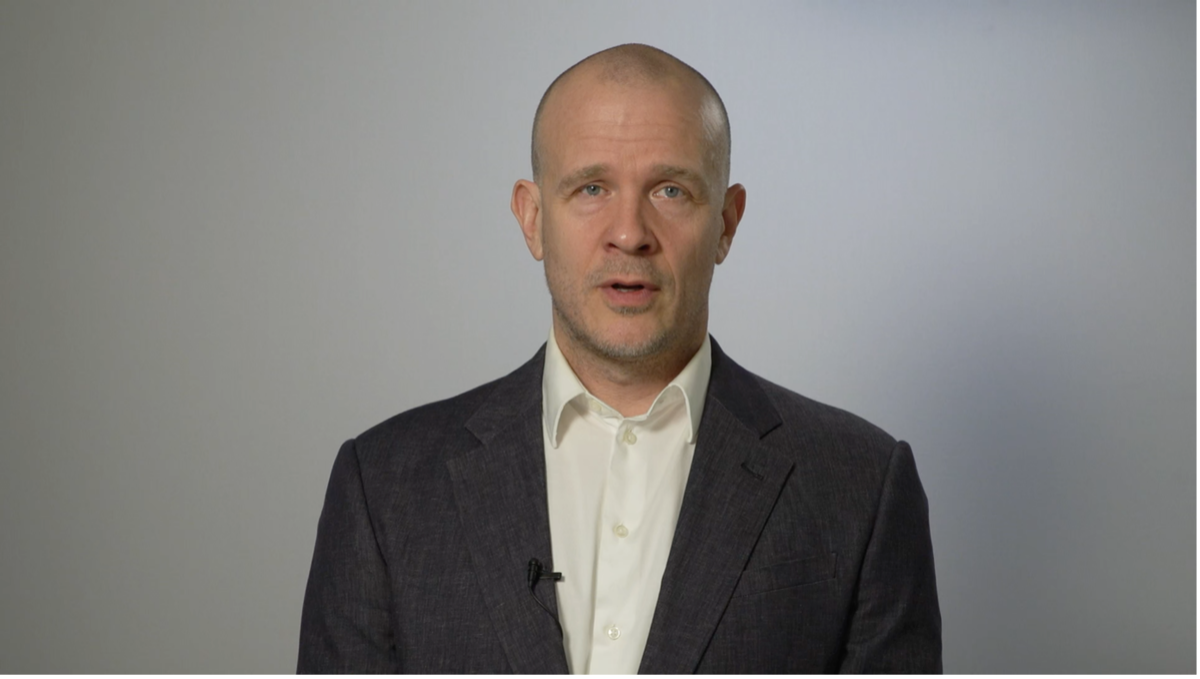
The thumbnail of the videos.

Based on the scripts, 12 videos were produced in collaboration with the Department for Development of Digital Learning Resources of the University of Stavanger. To avoid confounding factors, all other variables that the experimental ones were controlled for. We used the same professional actor to shoot all the videos, he wore the same clothes (white shirt and grey suit), and we had a neutral gray background for all videos (Figure 1). All videos were approximately the same length (range 54 s to 1 min 25 s). Videos are stored in the OpenScience repository of the University of Stavanger [46].

### Participants

The Norwegian Air Ambulance Foundation (NAAF), one of the Covcom project [45] partners, helped recruit participants among their member database. The member base of NAAF is made of donating members (who support the foundation through regular financial donations) and prospect members (who are interested and support in nonfinancial ways, eg, following and distributing content on their social media accounts). At the time of the data collection, there were approximately 230,000 donating members and 110,000 prospect members. As the former has been previously involved in research activities, we decided to recruit the study participants among the donating members. An a priori power analysis assuming multiple linear regression with 6 predictor variables, a small effect size of *F*=0.02, and 95% statistical power revealed that the minimum sample size was 1050 participants. From previous experiences, the response rates of NAAF members in previous research studies were approximately 10%. Therefore, we adopted a conservative approach and invited 12,000 people to participate, so that a response rate of 8.75% (1050/12,000) would be sufficient to reach the minimum sample size. Participants were randomly assigned to 1 of the 12 video versions and were invited by email. The email contained a brief explanation of the study and a link to participate.

Among the invited, 54% (6481/12,000) were male; ages ranged from 18 to 90 years, with a median value of 64.8 (IQR 58-73) years.

### Data Collection and Management

NAAF’s IT infrastructure was used to collect the data. This choice came with 2 advantages: first, participants would receive the email from NAAF, an organization they are familiar with, therefore lowering the risk of emails being perceived as spam. Second, as per General Data Protection Regulation (GDPR) requirements, no personal information had to be shared. The data were collected from June 1 to June 9, 2021.

Participants were sent an email containing 1 of the 12 videos, and a link to the survey created on the SurveyMonkey platform. Participants were randomly assigned to 1 of the 12 video versions. The data collection was anonymous: the IP addresses of respondents were not collected, and the email addresses were used only for inviting participants, without being stored or linked to responses. Respondents were not asked for any personal or sensitive data.

A unique identifier was generated for each response, and responses were stored in a database. This database is not linked to the NAAF’s members database.

### Ethical Considerations

Since the project did not intend to collect or process any personal or sensitive data, and because the data collection was anonymous (the IP address was not collected), the study was exempt from obtaining approval from the Norwegian Centre for Research Data or the Ethical Committee. In addition, the legal department of NAAF ensured that the study was compliant with the GDPR and national data processing regulations.

Participants gave their informed consent digitally at the beginning of the survey. Participation was voluntary and no incentives were offered to participants to complete the survey. Consent to use the image was granted from the actor depicted in Figure 1.

### Measures and Data Analysis

The questionnaire collected 6 individual characteristics: age, sex, education, income level, place of living, and belief in science. The Belief in Science scale [41] was used to define a baseline of the level of scientific belief of participants. The Belief in Science scale is a measurement tool of attitudes toward science, where science shares similarities with religion in terms of the comforting role it plays in individuals’ lives.

Comprehension is one of the main aims of health communication and was assessed as both subjective (or perceived) and objective comprehension. Here, subjective comprehension indicates the extent to which someone believes to have understood that information, while objective comprehension refers to the ability to understand the information and to incorporate it into one’s knowledge [47]. Subjective comprehension was measured by a 4-item, 5-point Likert scale, with internal consistency of the scale being assessed by the Cronbach α. Objective comprehension was measured by 1 single-choice question. There were 3 different questions that matched the message enclosed in the video. Participants were presented with 4 answer alternatives, of which 1 was correct. The English translation of the questionnaire is available in the Multimedia Appendix 1.

Multiple linear regression analysis was performed in R Studio (R Core Team) [48], with comprehension as the dependent variable and age, sex, education, income, place of living, and belief in science as independent variables. The model was also tested against generalized additive model analysis to account for nonlinear data such as the ceiling effect.

## Results

We collected 1194 complete replies from 12,000 invites, corresponding to a response rate of 9.95%. After removing 2 responses that failed the attention checks, a total of 1192 valid responses were included for analysis. This is greater than the threshold of 1050 subjects resulting from the power analysis.

The mean age of participants was 64.77 (SD 11.31) years, female individuals represented 46.14% (550/1192) of the total, and 53.1% (644/1192) lived in an urban context. Those with higher education (at least a college or university degree) comprised 53.69% (640/11921) and those earning more than NOK 500,000 per year comprised 41.86% (499/1192). The mean belief in science score, measured on a 1 to 6 scale, was 4.42 (SD 0.84). All values, including the details by video version, are available in Table 1 below.

**Table 1:** Characteristics of study participants (n=1192).

Group or version	Participants, n	Age (years), mean (SD)	Female individuals, n (%)	Living in city, n (%)	High education, n (%)	Income >NOR 500,000^a^, n (%)	Belief in science, mean (SD)
1	98	63.48 (11.64)	44 (44.9)	52 (53.06)	59 (60.2)	45 (45.92)	4.38 (0.88)
2	108	65.53 (12.04)	50 (46.3)	62 (57.41)	59 (54.63)	43 (39.81)	4.18 (0.85)
3	108	64.60 (10.45)	48 (44.44)	58 (53.7)	51 (47.22)	50 (46.3)	4.44 (0.77)
4	98	63.28 (11.61)	47 (47.96)	50 (51.02)	54 (55.1)	44 (44.9)	4.42 (0.80)
5	93	68.00 (10.77)	44 (47.31)	48 (51.61)	53 (56.99)	36 (38.71)	4.25 (0.88)
6	94	66.48 (11.08)	34 (36.17)	51 (54.26)	50 (53.19)	41 (43.62)	4.48 (0.66)
7	107	62.70 (11.84)	53 (49.53)	58 (54.21)	71 (66.36)	55 (51.4)	4.50 (0.77)
8	103	65.78 (10.2)	48 (46.6)	63 (61.17)	49 (47.57)	33 (32.04)	4.56 (0.84)
9	85	65.59 (10.05)	47 (55.29)	43 (50.59)	38 (44.71)	32 (37.65)	4.39 (0.87)
10	93	64.29 (14.79)	42 (45.16)	44 (47.31)	52 (55.91)	35 (37.63)	4.53 (0.92)
11	96	63.96 (10.35)	41 (42.71)	47 (48.96)	50 (52.08)	42 (43.75)	4.48 (0.82)
12	109	64.06 (9.85)	52 (47.71)	57 (52.29)	54 (49.54)	43 (39.45)	4.45 (0.90)
Total	1192	64.77 (11.31)	550 (46.14)	633 (53.1)	640 (53.69)	499 (41.86)	4.42 (0.84)

^a^Equivalent to USD $45,000, using a conversion rate of 1 NOK=0.09 USD.

We found that participants reported high levels of comprehension, as the mean for subjective comprehension was 5.48 (SD 0.63) on a 1 to 6 scale. Moreover, there was a limited degree of variation between video versions, with *z* scores ranging from –0.57 to 0.33. Regarding objective comprehension, we observed a mean value of 0.78 (SD 0.42) on a 0 to 1 scale, meaning that 78% (930/1192) of participants correctly understood the information in the video. In this case, between-groups variation was greater compared to subjective comprehension, with *z* scores ranging from –0.76 to 0.51. The complete overview is available in Table 2 below.

The data confirmed the internal consistency of the objective comprehension scale (α=.87).

**Figure 2:**
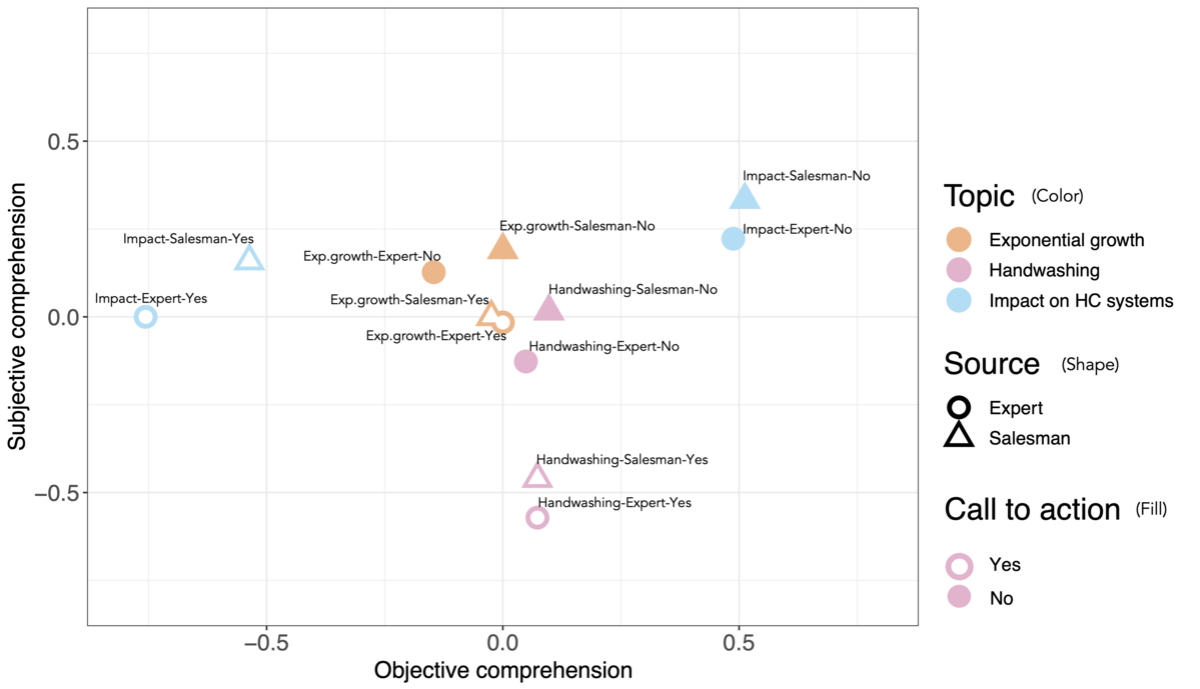
Objective versus subjective comprehension of the 12 video versions. The color displays the topic, the shape represents the source, and the filling shows the call to action. Exp.: exponential; HC: health care.

Figure 2 displays the levels of subjective and objective comprehension by topic, source, and call to action.

**Table 2:** Subjective and objective comprehension by video version.

Version	Subjective comprehension *z* scores	Objective comprehension *z* scores
1	–0.02	0.00
2	0.00	–0.02
3	0.13	–0.15
4	0.19	0.00
5	–0.57	0.07
6	–0.46	0.07
7	–0.13	0.05
8	0.02	0.10
9	0.00	–0.76
10	0.16	–0.54
11	0.22	0.49
12	0.33	0.51

An overview of the multiple linear regression analysis is presented in Table 3. We found that age had an impact on comprehension, with increased age being associated with lower comprehension, both subjective and objective. Moreover, the effect was larger on subjective comprehension than objective comprehension (β=–.13 vs β=–.07), and both effects were statistically significant (*P*<.001 and *P*=.02).

Our data revealed that sex had a statistically significant effect on both perceived and objective comprehension (*P*<.001 and *P*=.02) and that although effects were comparable in magnitude, they had different directions (Table 3). In fact, female individuals had a higher degree of subjective comprehension than males (β=–.10), while male individuals had a higher level of objective comprehension than females (β=.07).

The data showed that whether participants resided in an urban or rural area did not have any statistically significant effect on either subjective or objective comprehension (all *P*>.05).

**Table 3:** Overview of the multiple linear regression analysis.

Predictor	Subjective comprehension			Objective comprehension	
	Standardized β	*P* value	Standardized β		*P* value
Age	–.13	<.001	–.07		.02
Sex	–.10	<.001	.07		.02
Residence	.03	.37	–.03		.32
Education	.11	<.001	.14		<.001
Income	.07	.03	.07		.02
Belief in science	.23	<.001	<.01		.87

Instead, we found that education had a statistically significant effect on comprehension, where higher levels of education were associated with both higher subjective (β=.11; *P*<.001) and objective (β=.14; *P*<.001) comprehension. Similarly, we observed that income had a statistically significant effect on both subjective (β=.07; *P*=.03) and objective (β=.07; *P*=.02) comprehension.

Finally, the data showed that the belief in science had no significant effect on objective comprehension (*P*=.87), while it had a relatively high effect on subjective comprehension (β=.23; *P*<.001), meaning that the higher the belief in science, the higher the level of perceived comprehension.

These effect sizes and significance were also confirmed by generalized additive model analysis.

## Discussion

### Principal Findings

The results of this randomized controlled trial displayed mixed effects of individual characteristics on the comprehension of pandemic video communication, both confirming existing evidence and providing novel insights.

### Age

Our findings add evidence to the body of literature investigating how younger and older adults comprehend pandemic videos, as we found that increasing age is associated with lower comprehension, both perceived and objective. The older the people, the less they correctly understood the pandemic-related information presented in the video, and to an even greater extent, the less they perceived that they had comprehended that information.

This result is in line with previous research that suggests that attention should be given to age differences when developing pandemic communication videos [49,50].

### Sex

Sex was found to have a different effect on subjective and objective comprehension. While female individuals scored higher than male individuals on perceived comprehension, they scored lower on objective comprehension. In other words, males were less overconfident than females in their capacity to understand the pandemic-related information in the video. This result is surprising in light of previous evidence showing that men tend to be more overconfident than women [51,52]. A possible explanation is that the confidence gap between men and women varies with age. In a research study involving 8665 people, Zenger and Folkman [53] found that the confidence gap had an inverted trend in the mid-40s and widened after. After this age, women became more confident and overconfident than men. Given that the participants in our study mainly belong to the age group of 60 years or older, it is plausible that we observed the same trend as reported by Zenger and Folkman [53].

### Living Area

Rural or urban differences have been extensively reported in the literature in multiple disciplines, including several health domains [54,55]. We did not find any significant rural or urban difference in the comprehension of pandemic-related video information. This result is likely due to the structure of Norwegian society, as very little differences exist in terms of education and socioeconomic status (SES) between people living in rural and urban areas [56,57].

### Socioeconomic Status

The effect of education and income on the pandemic video communication outcomes has been investigated previously [19,37,58]. We found a significant and positive effect of education and income on comprehension, both perceived and objective. The higher the education and income level, the higher the likelihood of having higher levels of confidence in one’s ability to understand pandemic-related information (perceived comprehension) and to actually understand it (objective comprehension). Our findings add supporting evidence of the role that education and income play in social differences, encouraging policy makers to keep these differences into account when designing health communication strategies [59-61].

### Belief in Science

Results concerning the effect of belief in science on comprehension are contrasting: the higher the belief in science, the higher the perceived comprehension, while not leading to any statistically significant increase in objective comprehension. In other words, those with higher belief in science will understand as much as others, while they feel they understand more. A possible explanation for this finding could be rooted in the Dunning-Kruger effect, where individuals with higher confidence in their understanding may overestimate their actual knowledge [62]. This overconfidence might stem from their trust in scientific sources, which increases their perceived comprehension but does not necessarily reflect their actual understanding of the content. Prior research has shown that trust in science and scientific authorities can influence how individuals perceive and evaluate scientific information. People with higher beliefs in science may experience a confirmation bias, wherein they interpret information in a way that aligns with their preexisting beliefs and trust, thereby increasing their subjective sense of understanding [63]. Moreover, those with a strong belief in science might possess a higher level of scientific self-efficacy, which enhances their confidence in processing and understanding scientific information, leading to a higher perceived comprehension [64]. However, self-efficacy does not always correlate with actual performance [65], which might explain why their objective comprehension did not show a statistically significant increase.

### Implications

Our findings contribute to the growing, but still limited, extent of evidence on pandemic communication to support authorities in effectively delivering complex information to the public. Our main result is that one-size-fits-all pandemic video communication does not exist: the same message will be comprehended differently by the younger and the older adults, male and female individuals, those with lower and higher levels of education, those who earn less and those who earn more, and those who have a low and those who have a high belief in science.

The implication of this is that health authorities should make different versions of pandemic videos. As the older adults had lower comprehension than the younger adults, videos aimed at the former could be more explicative, simpler in language, and distributed more intensively and through different channels, to favor reach, viewing, and consequently comprehension [66-68].

In addition, in the context of limited resources and to maximize outcomes, more attention could be given to those with a lower SES. In addition to the previous recommendations, as in the Norwegian context, minority individuals often belong to the lower SES groups, do not speak Norwegian, and live in more closed communities, pandemic communication videos could be created (or subtitled) in their native languages [60].

These 5 characteristics (age, sex, education, income, and belief in science), which are a limited subset of all potentially significant individual attributes, indicate that authorities should to a much higher degree understand the different target groups and how these groups access and interpret information. This will then allow authorities to create tailor-made pandemic videos that can reach and effectively communicate information to the public.

At the same time, we should pay attention to the potential drawbacks of popularizing complex scientific information. As demonstrated by Scharrer et al [69], making science easier to understand for laypeople inclines them to underrate their dependence on experts. This is a concrete risk with increased saliency during pandemics as decisions are generally made under time pressure and based on partial and uncertain information.

### Directions for Future Research

Future research should explore targeted communication strategies that enhance comprehension among older adults since they will be an increasingly larger share of the population and have a relatively low level of understanding of the pandemic information presented in our videos. Future studies could test if videos using simpler language or interactive elements aid their comprehension of pandemic-related information. In addition, future research should investigate how confidence and comprehension in pandemic communication vary across different age and sex groups, and if the lack of urban-rural differences in comprehension holds true in other countries with more pronounced rural-urban divides. Moreover, developing and testing interventions aimed at reducing educational and income-related disparities in health communication comprehension is crucial, and we encourage researchers to address this matter. Furthermore, we need a deeper understanding of the mechanisms of belief in science and its impact on both perceived and objective comprehension; creating and testing interventions to align perceived and actual comprehension would enhance the understanding of these mechanisms. Finally, expanding the research to include a broader range of individual attributes beyond age, sex, place of living, education, income, and belief in science will help better tailor pandemic communication strategies to effectively reach and inform all segments of the population.

### Limitations

Our limitations include the choice of the sample, meaning that findings apply to a subset of the Norwegian population, those who are members of the NAAF, while no conclusion can be drawn about their generalizability to a national level. The mean age of our sample is higher than the mean age of the national population. Moreover, as donating members of the NAAF, we have reason to believe that their SES is higher than the general population. Additional country-specific factors such pose a challenge to the generalizability of our findings internationally. Moreover, the ceiling effect, and therefore little variation, in subjective comprehension levels might hide effects that we were not able to detect. Finally, the use of one single objective comprehension question is another limitation. Our choice was due to the absence of a validated scale that could be applied to all three topics communicated in our study.

### Conclusions

In this study, we investigated how the comprehension of pandemic-related video information is influenced by recipients’ characteristics. Our findings indicate that younger individuals understand and perceive their comprehension of pandemic information better than older individuals. Female individuals report higher levels of perceived comprehension, while male individuals exhibit higher levels of objective understanding. In addition, higher education and income levels are associated with greater comprehension, both objective and subjective. Belief in science plays an important role for perceived comprehension, while it does not have any effect on people’s ability to objectively comprehend pandemic-related information delivered through video.

These findings are relevant because effective communication during pandemics is vital for public health, ensuring that citizens are accurately informed and can take appropriate actions to protect themselves and others [70,71]. By highlighting the varying levels of comprehension across different demographic groups, our study underscores the need for tailored communication strategies that account for these differences. This approach is essential to maximize the reach and effectiveness of public health messages, particularly in diverse populations.

Our study contributes to the growing body of evidence on health communication [72-74] by providing insights into how different segments of the population understand pandemic-related information. It reinforces the notion that a one-size-fits-all approach is ineffective; instead, public health authorities should create multiple versions of pandemic videos to address the unique needs of various groups. These findings can guide future communication efforts, ensuring that critical health information is accessible and comprehensible to all citizens, ultimately enhancing public health outcomes during pandemics and other health crises.

## Appendix

Multimedia Appendix 1 Questionnaire.

## Abbreviations

GDPR: General Data Protection Regulation
NAAF: Norwegian Air Ambulance Foundation
SES: socioeconomic status

## Data Availability

The videos generated during and analyzed during this study are available in the OpenScience repository of the University of Stavanger [46].
