# Supplementary material for: Individual Characteristics in the Comprehension of Pandemic Video Communication: Randomized Controlled Between-Subjects Design
Source: J Med Internet Res. 2024 Dec 4;26:e48882. doi: 10.2196/48882 (PMC11656110; doi:10.2196/48882)
Supplement: Multimedia Appendix 1 [file jmir_v26i1e48882_app1.docx]

**Appendix**

Questionnaire (English translation)

*Dear participant, thank you for your contribution to this research. We appreciate you taking ten minutes of your time to help us. Watch the short video below. Then a few short questions will follow. Your answers are anonymous and will be analyzed in aggregate form. You will be updated on the findings as soon as the results will be available.*

1. The video you have seen was:

a. artistic

b. scientific

2. The speaker was:

a. a woman

b. a man

| **Statement** | Strongly disagree | Disagree | Slightly disagree | Slightly agree | Agree | Strongly agree |
| --- | --- | --- | --- | --- | --- | --- |
| 3. I understood the content of the video | 1 | 2 | 3 | 4 | 5 | 6 |
| 4. The message was clear | 1 | 2 | 3 | 4 | 5 | 6 |
| 5. I am able to recall the information | 1 | 2 | 3 | 4 | 5 | 6 |
| 6. I am able to tell/spread the message | 1 | 2 | 3 | 4 | 5 | 6 |

7a. If R=0.7, how many people will be infected by 100 positive?

a. 7

b. 30

c. 70

d. 170

7b. 5. For how many seconds should you wash your hands?

a. 5 seconds

b. 20 seconds

c. 1 minute

d. 2 minutes

7c. Why are pandemics dangerous for the healthcare systems?

a. because they are highly contagious

b. because if the system is overburdened, it cannot deliver standard care

c. because their lethality is high

d. because they can lead to lockdown measures

| **Statement** | Strongly disagree | Disagree | Slightly disagree | Slightly agree | Agree | Strongly agree |
| --- | --- | --- | --- | --- | --- | --- |
| 8. Science provides us with a better understanding of the universe than does religion. | 1 | 2 | 3 | 4 | 5 | 6 |
| 9. “In a demon-haunted world, science is a candle in the dark.” (Carl Sagan) | 1 | 2 | 3 | 4 | 5 | 6 |
| 10. We can only rationally believe in what is scientifically provable. | 1 | 2 | 3 | 4 | 5 | 6 |
| 11. Science tells us everything there is to know about what reality consists of. | 1 | 2 | 3 | 4 | 5 | 6 |
| 12. All the tasks human beings face are soluble by science. | 1 | 2 | 3 | 4 | 5 | 6 |
| 13. The scientific method is the only reliable path to knowledge. | 1 | 2 | 3 | 4 | 5 | 6 |
| 14. The only real kind of knowledge we can have is scientific knowledge. | 1 | 2 | 3 | 4 | 5 | 6 |
| 15. Science is the most valuable part of human culture. | 1 | 2 | 3 | 4 | 5 | 6 |
| 16. Science is the most efficient means of attaining truth. | 1 | 2 | 3 | 4 | 5 | 6 |
| 17. Scientists and science should be given more respect in modern society. | 1 | 2 | 3 | 4 | 5 | 6 |

18. How old are you?

19. Gender:

a. Woman

b. Man

c. I prefer not to answer

20. Do you live in a city?

a. Yes

b. No

21. Education:

a. Primary school (up to 10 years)

b. High school / vocational training (minimum 3 years)

c. College / University (less than 4 years)

d. College / University (4 years or more)

22. Your income is:

a. Less than 300.000 NOK

b. Between 300.000 and 500.000 NOK

c. More than 500.000 NOK

d. I prefer not to answer

23. If you would like to help us find out more and are willing to participate in future surveys, please register by filling out your email address.
